# The cancer testis antigens CABYR-a/b and CABYR-c are expressed in a subset of colorectal cancers and hold promise as targets for specific immunotherapy

**DOI:** 10.18632/oncotarget.27897

**Published:** 2021-03-02

**Authors:** H.M.C. Shantha Kumara, Elie Sutton, Otavia L. Caballero, Tao Su, Xiaohong Yan, Aqeel Ahmed, Sonali A.C. Herath, Vesna Cekic, Linda Njoh, Daniel D. Kirchoff, Richard L. Whelan

**Affiliations:** ^1^Division of Colon and Rectal Surgery, Department of Surgery, Lenox Hill Hospital, Northwell Health, New York, NY 10028, USA; ^2^Department of Surgery, Mount Sinai West Hospital, New York, NY 10019, USA; ^3^Ludwig Institute for Cancer Research Ltd., New York Branch of Human Cancer Immunology at Memorial Sloan-Kettering, New York, NY, USA; ^4^Herbert Irving Comprehensive Cancer Center, Columbia University, New York, NY 10032, USA; ^5^University of Vermont Medical Center, Internal Medicine Hospitalist Service, Burlington, VT 05401, USA; ^6^Department of Mathematics, City University of New York at Lehman College, Bronx, NY 10468, USA; ^7^Roper St. Francis Physician Partners Surgical Oncology, Charleston, SC 29403, USA; ^8^Donald and Barbara Zucker School of Medicine at Hofstra/Northwell, Hempstead, NY 11549, USA; ^9^Current address: Orygen Biotecnologia S.A., São Paulo, Brazil

**Keywords:** cancer testis antigens, colorectal cancer, CABYR a/b, c

## Abstract

Introduction: Calcium-binding tyrosine phosphorylation-regulated protein (CABYR) is expressed in the human germ line but not in adult human tissues, thus, it is considered a cancer testis protein. The aim of this study is to evaluate the CABYR isoforms: a/b and c mRNA expression in colorectal cancer (CRC) and to determine if these proteins hold promise as vaccine targets.

Materials and Methods: CABYR mRNA expression in a set of normal human tissues, including the testis, were determined and compared using semi-quantitative PCR. As regards the tumor and normal mucosal samples from study patients, RNA was extracted and cDNA generated after which quantitative PCR was carried out. Analysis of CABYR protein expressions by immunohistochemistry in tumor and normal colon tissues was also performed.

Results: A total of 47 paired CRC and normal tissue specimens were studied. The percent of patients with a relative expression ratio of malignant to normal (M/N) tissues over 1 was 70% for CABYR a/b and 72% for CABYR c. The percent with both a M/N ratio over 1 and expression levels over 0.1% of testis was 23.4% for CABYR-a/b and 25.5% for CABYR c. CABYR expression in tumors was further confirmed by immunohistochemistry.

Conclusions: CABYR a/b and c hold promise as specific immunotherapy targets, however, a larger and more diverse group of tumors (Stage 1-4) needs to be assessed and evaluation of blood for anti-CABYR antibodies is needed to pursue this concept.

## INTRODUCTION

Colorectal cancer is the third diagnosed cancer type as well as the second most common reason of cancer death in the United States, [1] however, in the last 30 years, there has been a substantial improvement in colorectal cancer associated mortality [2]. The five-year survival rate for patients diagnosed with colorectal cancer between 2003 and 2009 was 64.9% [3]. Although surgery remains the mainstay of therapy, new chemotherapeutic drugs and drug combinations have been introduced for use in both the neoadjuvant and adjuvant setting. Despite these accomplishments, at least 40% of patients who undertake “curative” colorectal surgery go on to develop metastatic disease [4]. Therefore, there continues to be a need for new anti-cancer agents and treatments.

The last 15 years has seen the development of immunotherapies for colorectal cancer. These advances include FDA approval of a number of monoclonal antibodies, which target epidermal growth factor receptor, vascular endothelial growth factor, and, most recently Programmed cell Death 1 protein (PD-1) or PD-1 ligand [5, 6]. Despite these advances as well as much research and effort, an effective tumor vaccine has not yet been developed. Regardless, investigators continue to search for new tumor cell target antigens that hold promise as vaccine targets.

Cancer Testis Antigens (CTAs), a subcategory of Tumor Associated Antigens (TAA’s), are a group of proteins that hold specific promise because they are expressed in the human germ line, but not in adult human tissues. CTA have mRNA expression that is restricted to the testis, fetal ovary, and placenta. Interestingly, up to 30–40% of a number of different cancer types (melanoma, bladder cancer, sarcoma, etc) express one or more CTAs [7]. As mentioned, because tumors express these proteins while normal adult tissues do not, select CTAs may have value as vaccine targets. The testis is an immune privileged location due to the blood-testis barrier. Therefore, the immune system is not familiar with proteins solely expressed in the testis. Thus, when CTAs are overexpressed in a tumor the immune system may develop a specific CD8+ T cell response to the protein in question. CTAs were first recognized by van der Bruggen et al. in the 1990; these investigators noted *in vitro* recognition of an antigen expressed by a human melanoma cell line gene. This gene did not show any similarity to known sequences and was not expressed in a panel of normal tissues [8].

Calcium-binding tyrosine phosphorylation regulated protein (CABYR) is a protein localized to the fibrous sheath of the flagellum of spermatozoa, and it exhibits calcium binding when phosphorylated during capacitation. Five isoforms of this protein have been identified: CABYR a, b, c, d, and e [9]. Luo et al. demonstrated CABYR expression in lung cancer cells and have identified it as a Cancer-Testis antigen. By performing real-time PCR to determine expression levels of CABYR a/b and c in the tissues of lung cancer patients, they were able to find expression in 36% and 42% of lungs cancer tissues, respectively. Expression was confirmed with positive staining for CABYR on Immunohistochemistry (IHC). Interestingly, anti-CABYR antibodies to CABYR a/b and c were also noted in the sera of those patients [10]. CABYR c and -d are also expressed in brain tumors, and to a lesser extent normal brain tissue. Upon discovery of this information, it was thought that CABYR might not be a cancer testis antigen [11]. However, this does not disqualify it as a CTA, as the brain, like the testes, is an immune privileged site, protected by the blood-brain barrier. CTAs that are restricted to the testis and the brain are known as testis/brain restricted CTAs, and 14 such proteins have been identified [12]. Additionally, CABYR c is highly expressed in hepatocellular carcinoma tissues and may play an oncogenic role in hepatocarcinogenesis [13], Thus far, the expression of CABYR in colorectal tumors has not been previously studied. In the present study, expression levels of CABYR a/b and c are compared in 47 paired colorectal tumor and normal colonic tissue specimens.

## RESULTS

### Demographics and clinical data

A total of 47 patients who underwent elective colorectal cancer resection were included in this study. The mean age of the patients was 65 ± 16.8 years (38% male, 62% female). The tumor locations were as follows: right, 25 (53%); sigmoid/rectosigmoid, 10 (21%); rectal, 6 (13%); and left or transverse, 6 (13%). The final stage distribution was: Stage II, 26 pts (56%); Stage III, 19 pts (40%); and Stage IV, 2 pts (4%). No patients received neoadjuvant pelvic radiation and chemotherapy.

### Expression of CABYR a/b and CABYR c

The expression of CABYR a/b and c in 22 normal adult tissues were evaluated using semi-quantitative RT-PCR to be able to interpret the background expression values.

**Figure 1:**
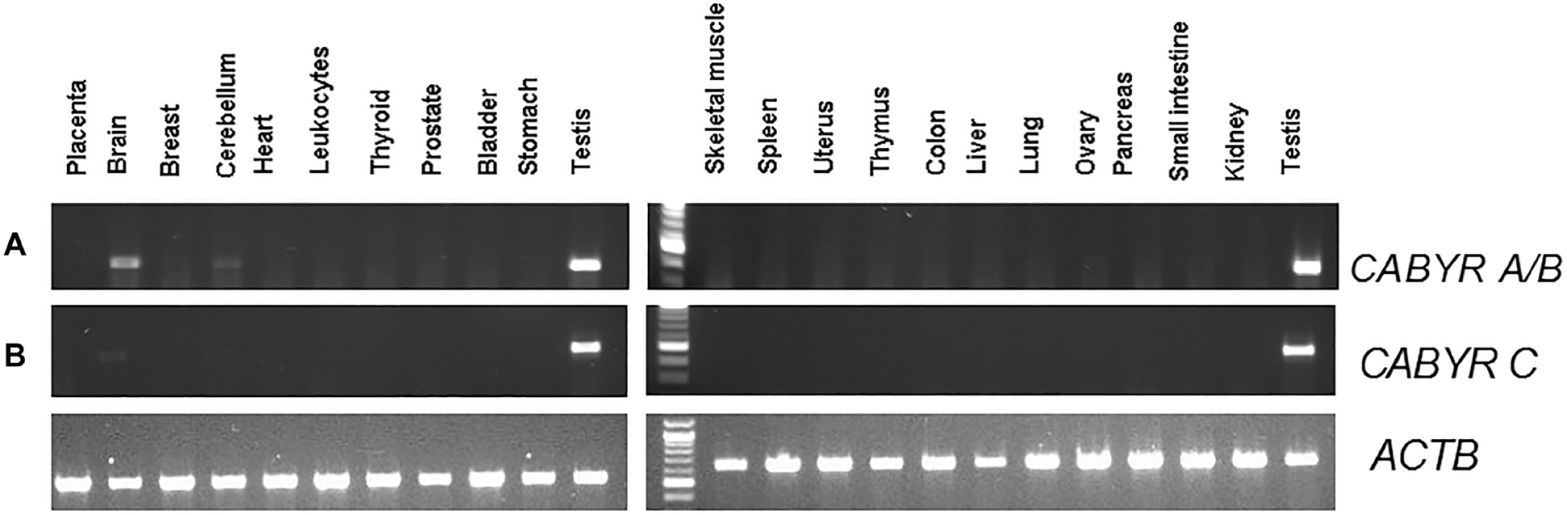
(**A**) Semi-quantitative analysis of CABYR a/b expression in pooled RNA various different normal human tissues (testis, placenta, bladder, brain, breast, cerebellum, colon, small intestine, heart, kidney, leukocytes, liver, lung, skeletal muscle, ovary, pancreas, prostate, spleen, stomach, thymus, thyroid, uterus). (**B**) Semi-quantitative analysis of CABYR c expression in pooled RNA various different normal human tissues (testis, placenta, bladder, brain, breast, cerebellum, colon, small intestine, heart, kidney, leukocytes, liver, lung, skeletal muscle, ovary, pancreas, prostate, spleen, stomach, thymus, thyroid, uterus). ATCB band in each lane was human β-actin (ACTB) amplified as control for each tissue types.

CABYR a/b was expressed in the testis and weakly expressed in the brain, without significant expression in any other normal tissues. CABYR c expression was observed only in testis tissues among 22 normal tissues tested (Figure 1A and 1B).

All 47 samples of tumor and normal tissues (18 male, 29 female) were analyzed for CABYR a/b and c expression via quantitative PCR. The threshold for positive expression was established as to be a response greater than 0.1% of testicular expression levels.

**Figure 2:**
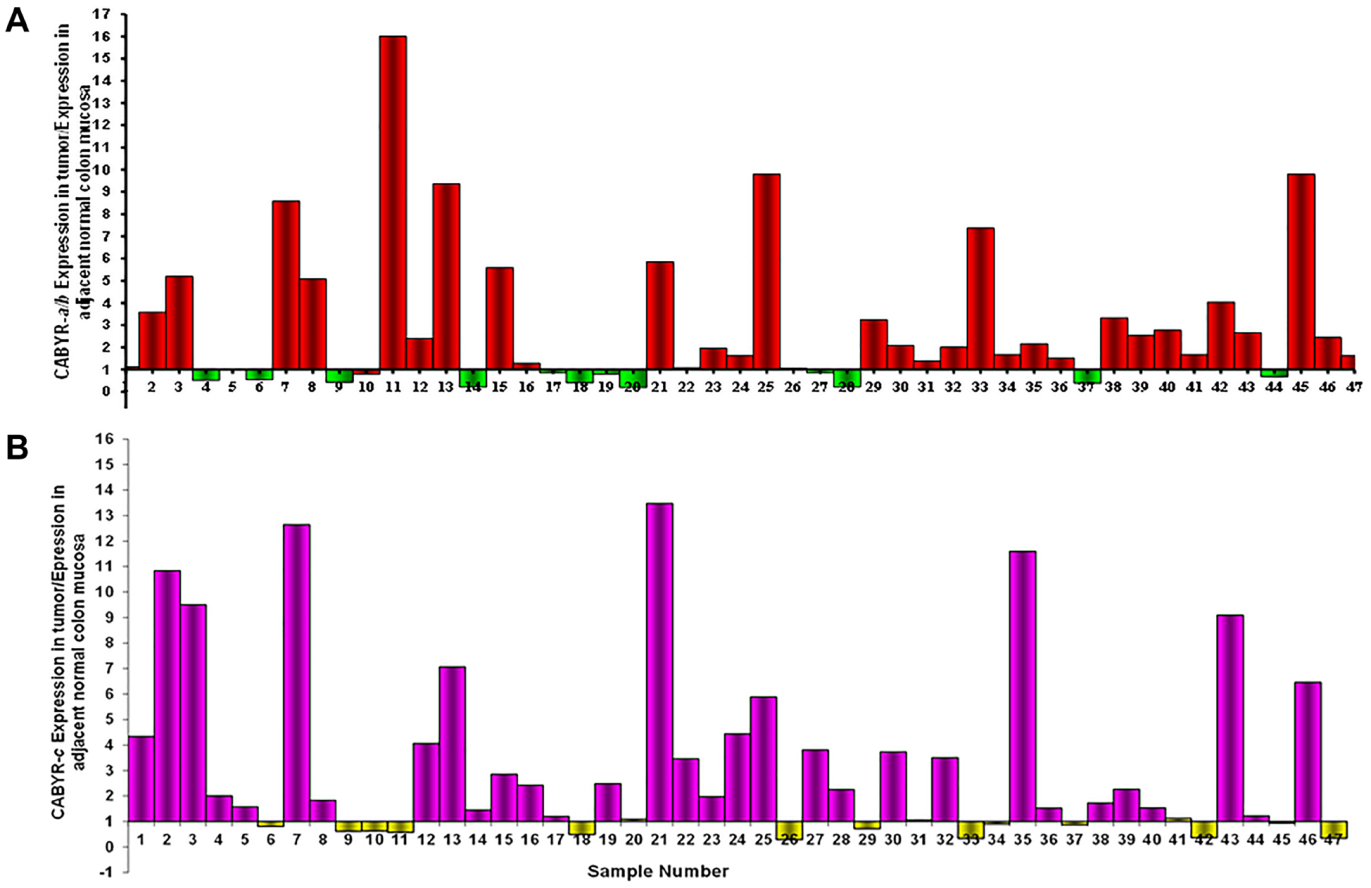
(**A**) CABYR a/b relative expression tumor vs. adjacent normal mucosa. (**B**) CABYR c relative expression tumor vs. adjacent normal mucosa.

**Figure 3:**
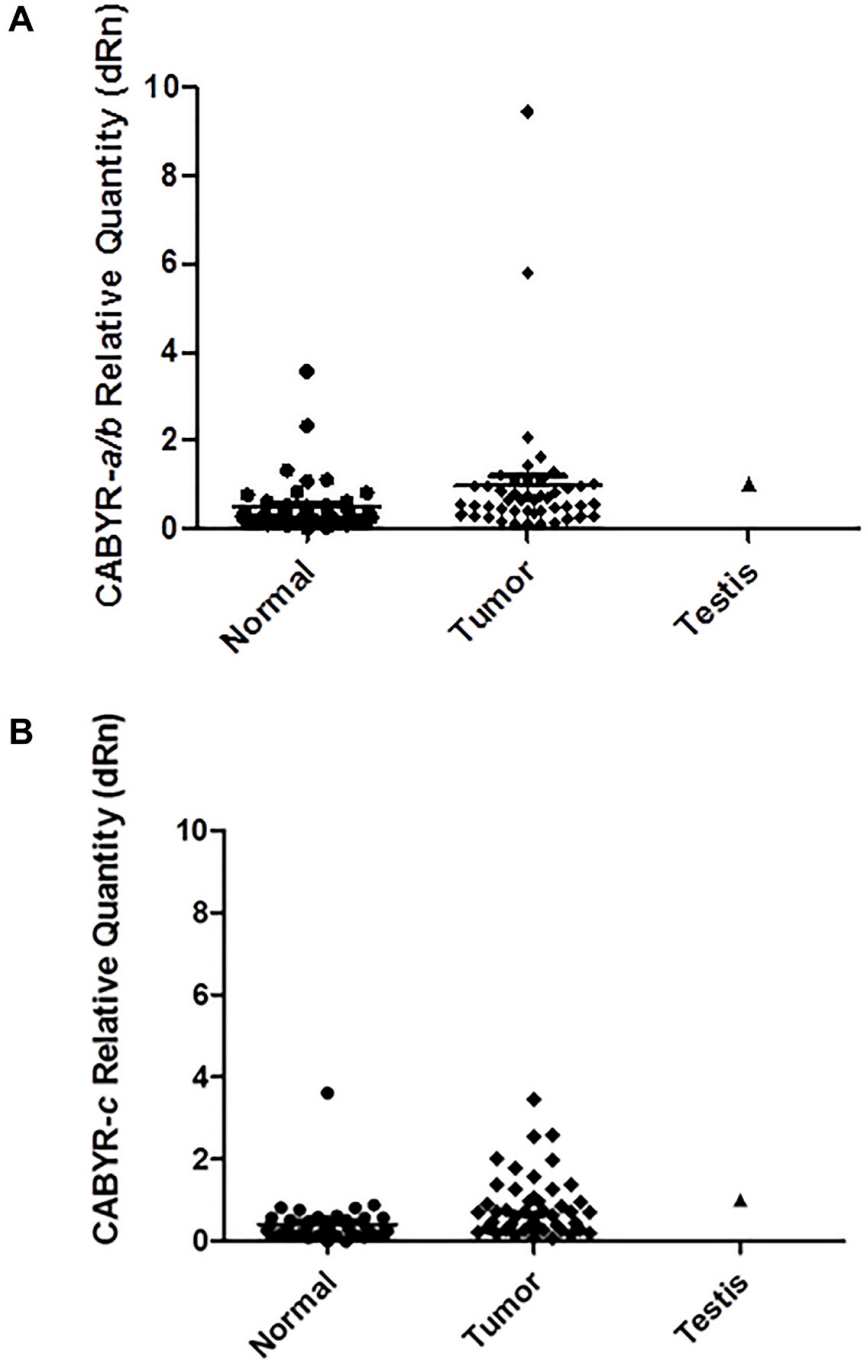
(**A**) CABYR a/b relative quantity (dRn) [normalized to Testis = 1). (**B**) CABYR c relative quantity (dRn)[normalized to Testis = 1).

**Figure 4:**
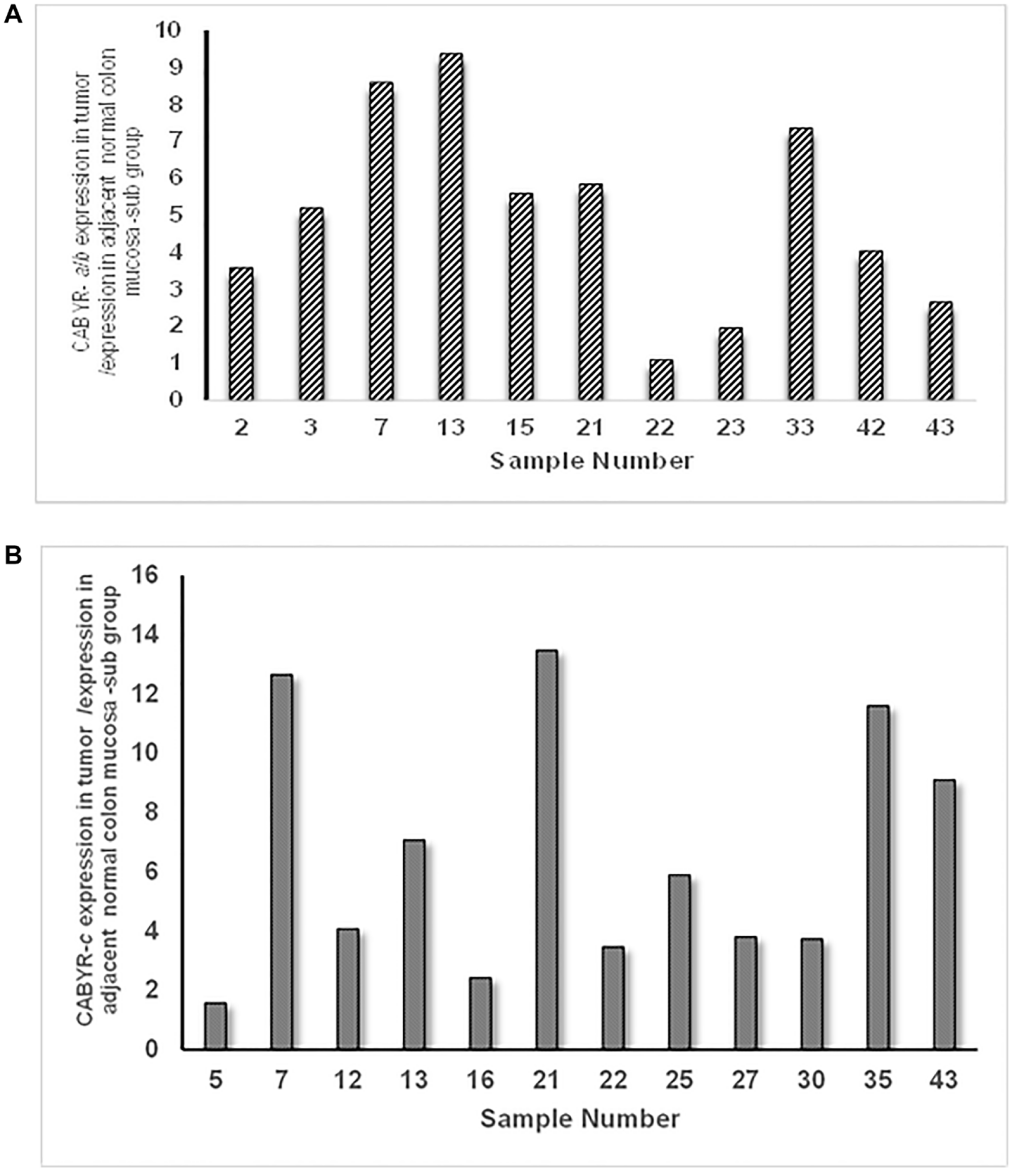
(**A**) Sub group of tumors with relative expression of CABYR a/b Tumor vs. adjacent normal mucosa over 1 and expression levels over 0.1% of testis. (**B**) Sub group of tumors with relative expression of CABYR c Tumor vs. adjacent normal mucosa over 1 and expression levels over 0.1% of testis.

A relative expression ratio of malignant to normal tissues over 1 was noted in 70% of paired samples for CABYR a/b and in 72% of paired tumor/normal tissue sets for CABYR c (Figure 2A and 2B, Figure 3A and 3B). Both expression ratios over one (1) and expression levels over 0.1% of testis was noted for CABYR a/b in 23.4% of tumor samples and for CABYR c in 25.5% of samples (Figure 4A and 4B).

The relative expression levels of CABYR a/b ranged from 0.2–5 times in 40/47 tumors and from 5–9 times in 2/47 tumors. Likewise, the relative expression levels of CABYR c ranged from 0.2–5 times in 37/47 tumors and from 2-33 times in 3/47 tumors.

### Immunohistochemical evaluation

**Figure 5:**
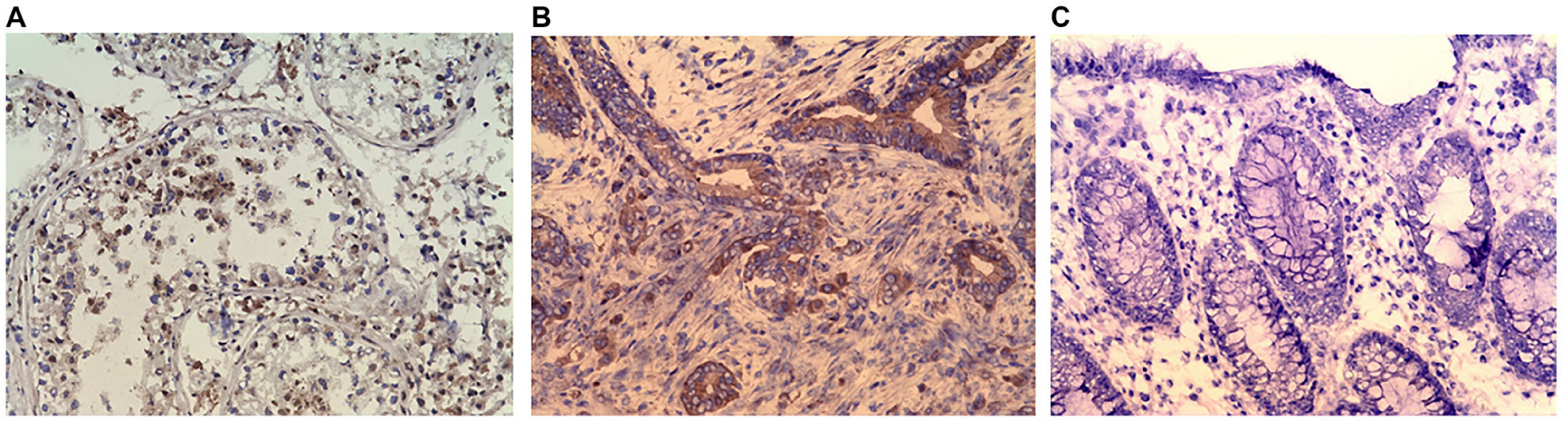
(**A**) Immunohistochemistry (IHC) staining of testis (positive control). (**B**) IHC staining of colonic adenocarcinoma. (**C**) IHC stating of Normal colonic mucosa (negative control). Purified rabbit anti-human CABYR polyclonal antibody for CABYR a/b and c antigen isoforms were used in IHC.

Immunohistochemical evaluation was conducted confirm the CABYR expression in sub set of tumors. Initially, IHC analysis was undertaken in 40 pairs of tumor/normal colon available for the study. Eighteen of the 40 frozen tumor sections assessed (45%) had positive CABYR staining in > 10% of neoplastic cells whereas 6 tumor sections had positive CABYR staining in < 10% of neoplastic cells (15%). The remaining 16 tumors were negative for IHC staining. Figure 5A depicts a section of normal seminiferous tubule having positive nuclear staining for CABYR antigen on germ cells. Figure 5B depicts stained colonic adenocarcinoma having 3+ positive nuclear staining for CABYR antigen, and Figure 5C depicts a section of adjacent normal colonic tissue having negative staining for CABYR antigen.

Non-significant increases in CABYR a/b expression levels were noted in Stage 2 (0.86 ± 1.1) and Stage 3 (1.11 ± 2.1) tumor samples; a similar trend was noted regarding CABYR C expression in Stage 2 (0.93 ± 0.85) and Stage 3 (2.47 ± 7.3) tumors. Furthermore non-significant increase of mean CABYR a/b and c expression were noted in node positive group vs. node negative specimens.

## DISCUSSION

Identification and characterization of new CT antigens is of great value as regards to the development of cancer immunotherapies. CABYR is a recently identified protein and very little is known about it, aside from a basic description and its role in sperm capacitation. As mentioned, it is currently known that isoform expression is present in brain tumors, hepatocellular carcinoma, and lung tumors. Nothing is known yet about this proteins role, if any, in the progression of these cancers. At the time this study was undertaken, there was no previously documented study of CABYR a/b and c in the setting of CRC. The purpose of this study was to determine CABYR expression in human CRC and to determine if this protein holds promise as a possible vaccine target for large bowel adenocarcinomas.

Unlike monoclonal antibodys which are passive immune therapies directed against key tumor proteins, the goal of tumor vaccines is to generate a cell mediated immune response (CD8+ T cells) against the tumor. It is hoped that the discovery of novel tumor associated antigens will lead to the development of tumor vaccines that could be used in colorectal cancer patients both in the pre- and postoperative setting. Ideally, these vaccines would elicit a specific CD8+ T cell response [14]. Preoperative vaccines would, hopefully, shrink tumors prior to surgery but should also provide patients postoperatively with a means of combatting tumor cells or micrometastases that remain. Marchand et al reported a 28% response rate in HLA-A1 positive melanoma patients with advanced disease who were given a MAGE-3 nonapeptide vaccine monthly for 3 months [15].

In this study, 47 paired colorectal tumor and normal tissue samples were assessed. Because of the similarity of the nucleotide sequences of CABYR a and CABYR b it is difficult to distinguish the two proteins, for this reason, the PCR assay that has been developed detects both isoforms (CABYR a/b) by utilizing primers that amplify a shared segment of nucleotides. In this study, tumor expression of both CABYR a/b and CABYR c was determined by quantitative PCR following RT-PCR.

As regards mRNA, notable CABYR a/b and c expression was noted in tissue samples of colon cancer and normal testis/placenta. In addition, these proteins were weakly present in the brain but not in the other 18 types of normal tissue that were evaluated.

As mentioned, IHC was also carried out to assess for the presence of CABYR proteins in colon cancer. It was noted that CABYR protein was, in fact, frequently expressed in many of the colon cancer samples that were analyzed. In contrast, CABYR protein was not detected in normal colonic mucosa. These IHC results, therefore, confirmed the mRNA results.

Unfortunately, the current investigators were not able to analyze preoperative plasma or serum from the CRC patients for the presence of auto-antibodies to CABYR. Determining the *in vivo* immunogenicity of CABYR is the logical next step when assessing a possible target antigen for a vaccine. Confirming the presence of a spontaneous immune response by finding auto-antibodies in the blood would suggest that this protein is capable of eliciting an immune response and that CABYR may have value as a vaccine target [16]. To further assess CABYR as a target antigen for a vaccine, blood samples from pre-resection CRC patients should be screened for auto-antibodies. Despite the lack of understanding of this protein’s function, its presence in colon cancer cells is sufficient to warrant further study regarding its potential as an immunotherapeutic target.

It should be noted that anti-tumor vaccines utilizing CT proteins have successfully been made and tested. As an example, MAGE-A3, a CT protein, has been approved as adjuvant immunotherapy in the treatment of NSCLC in patients whose tumors express MAGE-A3. Interestingly, despite the fact that antibodies against MAGE-A3 were observed in only 10% of patients at baseline, this vaccine was associated with a 25% relative reduction of relapse [17]. These results demonstrate that an immune response against the tumor can be generated by a vaccine despite the absence of pre-existing antibodies to the CTA in question.

A major concern regarding CT protein studies is that the source of gene expression is still unknown. Regulation factors are currently being investigated and many theories have been proposed for multiple gene family lines but have still yet to be proven. One such theory is activation of gametogenic tendencies present in master genes due to mutational events. Immunohistological testing has provided support by showing the development of CTA expression in tumor tissue that is similar to that noted in early testis development suggesting intervention or “switching on” of a suppressed master gene by mutational events [18].

As regards the shortcomings of this study, the lack of plasma or serum auto-antibody data was already mentioned. Other shortcomings of the study include the fact that while this study did include tumor samples from CRC stages 2–4 there were only two samples from stage IV patients and none from stage I patients. Ideally, reasonable numbers of specimens for each cancer stage would be assessed; the need for Stage 4 tumors is especially important to determine if CABYR expression, in general and for the individual isoforms, correlates with disease stage. Presently, because of the relatively small number of samples studied it is not possible to state whether CABYR expression correlates with tumor stage. Finally, this report supplies no oncologic outcome data (recurrence and survival rates) for the patients included in this study.

## MATERIALS AND METHODS

### Study population

Patients for this study was Pre-screened and selected as described by Shantha Kumara et al. [19–21]. Briefly total of 47 patients diagnosed with colorectal cancer who underwent elective colon/rectal resection were eligible for this study. All patients were enrolled in an IRB approved prospective clinical, demographic data and tissue bank. Those who had an adequate volume of frozen tumor and normal colonic tissue were available, were included in this study. This tissue and data banking protocol was organized by the New York-Presbyterian Hospital, Columbia University Health Science Campus in New York. Tumor and normal colon samples from periphery were obtained from operative specimens after gross evaluation by a pathologist. Normal tissue samples obtained from a distance from the tumor periphery along with each tumors. Tissue samples were flash frozen in a timely fashion after which they were maintained at −80°C until the time of analysis. Additional samples of the collected tissues were preserved for paraffin blocks. Patient’s demographic data, the location of the colon cancer, the indication for surgery and the final pathological staging results, were prospectively gathered for study subjects. As per protocol immunocompromised or recently transfused patients were not eligible for the study.

### Tissue harvest and preparation

Tissue samples preparation was carried out as explained previously [20, 21]. Tumor and grossly normal samples of mucosa taken from an area at least 2 cm away from the tumor. Tissue samples were harvested from operative specimens after a pathologist had assessed the gross specimen. Tissue samples were positioned in separate commercially available Cryomolds (Tissue Tek, Sakura Finetek, Torrance, CA, USA) which were then filled with OCT (optimal cutting temperature) compound (Tissue Tek, Sakura Finetek, Torrance, CA, USA) and placed in liquid nitrogen for snap freezing. The solid frozen tissue samples were stored in a −80°C freezer until further analysis.

### Initial tissue evaluation

Tissue samples were evaluated prior to being included in the study as explained by Shantha Kumara et al. [19]. Briefly selected fresh frozen blocks from the tissue bank were sectioned at the macromolecular laboratory at the Irving Comprehensive Cancer Center, Columbia University, New York. Identified frozen tumor and normal colonic tissue sections were taken at −20°C were then assessed histologically to confirm the quality and presence of tumor of each sample. After confirmation by pathologist, 30 sections of 10 μm thickness were collected in PCR tube for RNA extraction. Further, 10 sections of 5 μm thickness were harvested from same tumors and used for Immunohistochemistry.

### RNA extraction

RNA extraction from PCR tube sections were carried out as described by Shantha Kumara et al. (2012). Total RNA was isolated from the tissue samples using Qiazol (Qiagen, CA, USA) and purified with miRNeasy Kit (Qiagen, CA, USA). Briefly, OCT frozen tissue section were placed into a 2 ml sterile DNase and Ranse free Eppendorf tube, and then homogenized using TissueLyser II (Qiagen, CA, USA) in 1 ml Qiazol medium. The aqueous phase was recovered from chloroform-derived phase separation and used for acid phenol-chloroform extraction using a phase lock gel tube (Qiagen, CA, USA) to recover total pure RNA according to kit instructions. RNA quality was confirmed by agarose gel electrophoresis. The concentration of RNA in each samples were determined by measuring OD260nm via BioPhotometer (Eppendorf, NY, USA).

### Reverse transcription

The cDNA synthesis first strand reverse transcription was carried out using the ABI High Capacity RNA-to-cDNA Kit (Applied Biosystems, CA, USA) following the method described by Shantha Kumara et al. [21]. Briefly, 1 μg of total RNA was dissolved in 10 μl of 2X reverse transcription buffer, and 1 μl of 20X enzyme in a total volume of 20 μl and the reaction mixture was then incubated at 37°C for 60 minutes, followed by 95°C for 5 minutes, and held at 4°C. The synthesized cDNA was stored at −20°C until further analysis.

### Quantitative PCR

Comparative quantitative PCR was carryout using the method described by Shantha Kumara et al. [19–21]. Briefly, 300 nM forward and reverse primers, and 10 ng cDNA (corresponding to the amount of input RNA) were mixes in 20 μl volume and with a final concentration of 1X reaction buffer to make the final PCR reaction mixture. The sequence of forward and reverse primers used for CABYR a/b and c are described in Table 1. The following PCR reaction steps were used; hot-start at 95°C for 15 minutes, and then 45 cycles of 95°C for 20 seconds, 55°C for 30 seconds, and 72°C for 30 seconds after which a dissociation curve measurement from 55 to 95°C was performed. Template control (NTC) was not included in every assay and all samples were done in duplicate. The data analysis was carried out using software MxPro (Stratagene, CA, USA). Comparative quantitative analysis was carried out based on delta-delta Ct method using GAPDH as internal control. Results are reported as relative quantity (dRn). dRn is the magnitude of the fluorescence signal generated during the PCR at each time point that normalized to the reference dye after subtraction of background signal. Each plate was included with amplification on testis and placenta cDNA template as intra-/cross-plate calibrator. The analysis was carried out using the Mx3005P real-time PCR machine (Stratagene, CA, USA), using the QuantiTect SYBR Green PCR kit (Qiagen, CA, USA).

**Table 1:** CABYR *a/b* and *c* primer information

Primer ID	Primer name	Sequence	Start	Tm	Location	Product size	SNP
510	CABYRab-CF	GCAGTCACCACGAGTTAGTCC	1163	62.85	exon4	510	none
511	CABYRab-CR	CCTCGTTCACTTGTTGCCAT	1672	60.95	exon5		none
512	CABYRc-DF	ACTTACTATGTATAGAGGGAATACTAC	189	57.45	exon2-3	431	none
513	CABYRc-DR	GTTCACTTGTTGCCATTGCTAA	619	60.22	exon4-5		none

### Semi-quantitative RT-PCR

The expression of CABYR a/b and c in 22 normal adult tissues were analyzed as described by Shantha kumara et al. [21]. Briefly, pooled RNAs from normal tissues (testis, placenta, bladder, breast, brain, cerebellum, colon, heart, kidney, leukocytes, liver, lung, ovary, pancreas, prostate, small intestine, skeletal muscle, spleen, stomach, thymus, thyroid, uterus) were obtained from Clontech laboratories, Inc. (Palo Alto, CA, USA) and Ambion, Inc. (Austin, TX, USA). Reverse-transcription PCR was conducted using 1 μg of commercial RNA from each normal tissues and oligonucleotide (dT)12-18 primers in a total volume of 20 μl reaction mixture following the manufacturer’s protocol. The Omniscript RT kit (Qiagen, Valencia, CA, USA) and RNaseOUT reagent(Invitrogen, Carlsbad, CA, USA) were used. The synthesized cDNA was diluted five times with nuclease free water, and 3 μl of diluted cDNA were used in total volume of 25 μl PCR reactions. JumpStartTMREDTaq RedyMix (Sigma Aldrich, St.Louis, MO, USA) and 10 pmol of each primer were used for amplification. Primers used for PCR amplification were developed using Primer3 software (http://frodo.wi.mit.edu/cgi- bin/primer3/primer3_www.cgi) [Primer sequences; CABYR a/b- forward primer: GCAGTCACCACGAGTTAGTCC, reverse Primer: CCTCGTTCACTTGTTGCCAT; Amplicon size: 510bp, CABYR c- forward Primer: ACTTACTATGTATAGAGGGAATACTAC, reverse Primer: GTTCACTTGTTGCCATTGCTAA; Amplicon size: 431 bp].

Primers were developed to have annealing temperature around 60°C in order to include introns, thus allowing product discrimination in the case of genomic DNA amplification. Primer specificity was confirmed by aligning with the NCBI sequence database using BLAST http://blast.ncbi.nlm.nih.gov/Blast.cgi. ß-Actin (ACTB) was amplified as control using the following primers: forward primer: TTCTACAATGAGCTGCGTGTGGC, reverse primer: CTCCAGGGAGGAGCTGGAAGCA, amplicon size: 444 bp. The PCR amplification program used was following; pre-cycling hold at 95°C for three minutes followed by 35 cycles of denaturation at 95°C for 15 seconds, annealing for 30 seconds (10 cycles at 60°C, ten cycles at 58°C and 15 cycles at 56°C) and extension at 72°C for 30 seconds followed by a final extension step at 72°C for 7 minutes. PCR products were analyzed via electrophoresis on 1.5% agarose gels stained with ethidium bromide.

### Tumor tissue selection for IHC

Tumor tissue selection was carried out as described by Shantha Kumara et al. [19–21]. Forty [40] OCT frozen tumors and paired normal mucosal tissues selected for study were used to obtain 5 μm-thick tissue sections. Tumor sections without damages were then stained with Hematoxylin and Eosin (H&E) and were reviewed by two pathologists independently to confirm the presence of malignant tissue and proportion of tumor and noncancerous tissue in each section. Similarly, the 40 normal colon tissue sections were also assessed after H&E staining.

### IHC analysis

Immunohistochemistry analysis was carried out on 80 frozen slides (40 tumor/normal pairs selected for the study) using standard IHC protocol which was described by Shantha Kumara et al. [19, 20]. Fresh frozen sections obtained from normal testis and pancreatic adenocarcinoma tissues were used as positive controls. 5 μm thick tissue sections obtained from the frozen blocks were stained with H&E. IHC staining was performed on pre assessed 5 μm-thick sections of fresh frozen samples using purified rabbit anti-human CABYR polyclonal antibody for CABYR a/b and c antigen isoforms (Protein Tech Group Inc, IL, USA). The endogenous peroxidase activities of sections were blocked by immersing slides in H2O2 solution for 10 minutes at room temperature. Antigen retrieval was carried out by steamer heating method in 10 mmol/L citrate buffer (pH 6.0). After epitope recovery, slides were then incubated with 1:250 diluted primary CABYR antibodies overnight at 4°C. Slides were washed and incubated with 1:500 diluted secondary biotinylated anti-mouse IgG (H+L, Vector Laboratories, Inc.) and tertiary streptavidin-peroxidase conjugate (ABC complex, Vector Laboratories, Inc.). Slides were finally treated with chromogen “diaminobenzidine” for antigen detection. Counterstaining of tissue sections was performed with hematoxylin. Finally dehydrated, cleared in xylene, and were mounted on coated slides for evaluation.

### Evaluation of immunohistochemical staining

Immunohistochemical evaluation was performed independently by two pathologists who assessed by based on percentage of immuno-stained CABYR expressing cells in tissue sections. IHC staining was considered “positive” when at least 10% or more of the neoplastic cells stained positively. Furthermore, the intensity of staining was scored on a scale of 1 to 3 (1+: weak, 2+: moderate, and 3+: strong). IHC staining of 1+ intensity in < 10% of neoplastic cells was scored as negative, whereas IHC staining of 2–3+ intensity in < 10% of cells was scored as indeterminate.

### Statistical analysis

Data analysis was conducted following methods reported by Shantha Kumara aet al previously [19–21]. In short, the demographic and clinical data are reported as follows; the continuous variables were reported as mean and standard, while categorical variables were reported as frequencies and percentages. Statistical comparison of continuous variables was carried out by the *t*-test. The nonparametric Spearman’s Rho test which tests for correlation between two rank-ordered variables was used to find the relationship between multiple ordinal variables with non-normal distribution. The association between CABYR a/b and c expression levels of different subgroups such as nodal positive vs. nodal negative and overall cancer stage were assessed by Mann–Whitney test. A *p* value < 0.05 was considered as statistically significant.

## CONCLUSIONS

In conclusion, this study assessed colorectal cancer specimens for CABYR a/b and c expression. RT-PCR analysis demonstrated prominent expression of CABYR a/b and c in most CRC tumors when compared to adjacent normal colon tissue. The relative expression ratio of malignant tissue to normal tissues was greater than 1 in 70% of tumors for CABYR a/b and 72% of tumors for CABYR c. The percent with both malignant tissue to normal tissue ratio over 1 and expression levels over 0.1% of testis was 23.4% for CABYR a/b and 25.5% for CABYR c. CABYR expression was further confirmed by IHC in a subset of samples. The data of this study suggests a larger and more diverse group of tumors (Stage I–IV) needs to be assessed to determine if CABYR expression correlates with T, N, or final tumor stage. Furthermore, blood samples from CRC patients need to be evaluated for anti-CABYR antibodies. CABYR shows some promise as a vaccine target for a subset of CRC patients; further study is warranted.

## Abbreviations

CABYR: Calcium-binding tyrosine phosphorylation regulated protein
CTA: Cancer Testis Antigens
TAA: Tumor Associated Antigens
PCR: Polymerase chain reaction
RT-PCR: Reverse transcription polymerase chain reaction
ACTB: Human β-actin
